# Women’s Medical Society of N. Y. State

**Published:** 1912-06

**Authors:** 


### Women's Medical Society of N. Y. State

THE Sixth annual meeting of the Women's Medical Society of New York State took place in the banquet hall of the Hotel Statler on Friday, May 10, 1912. The Society was called to order by the President, Dr. Grace Peckham Murray. After the invocation by the Honorary President, Dr. Eliza M. Mosher of Brooklyn the address of welcome was given by Dr. Jane Wall Carroll of Buffalo. The reports of special committees was followed by the President's Address on "The present status of Women in Medicine" in which she showed the great improvement in the position of women today compared with the struggles and difficulties that had to be overcome by the pioneer women in the early days of their admission to the profession, that today they had nearly as many advantages as their brothers in the field. She regretted however, that there seemed to be a falling off in the numbers entering the profession, that there was a tendency more towards a commercial life. She suggested that young college graduates should be urged to enter the medical profession as there was much missionary work and salaried positions awaiting them, and she hoped they could be persuaded to do so.

A very interesting paper was given by Dr. Edith R. Hatch on "Acute Pyelitis in Infancy" showing the frequency in females, rarely in males, and usually due to the colon bacillus. Errors in diagnosis. Importance of examining the urine in obscure high temperatures. Methods of collecting urine. Treatment.

Dr. Esther Parker of Ithaca gave "Facts Concerning the Physical Condition of Women during College Life" under four heads. 1—General Nutrition. Condition at entrance compared with later condition. Neuroses. 2—Menstruation. Irregularities. Dysmenorrhoea. Amenorrhoea. Nervous disturbances. 3—Thyroid enlargement. 4—Result of Gymnasium work.

Dr. Ida C. Bender, took up the "Present Status of teaching Hygiene in the School.

Dr. Cora Billings Lattin gave the "Report of the State Chairman of the American Medical Association, Public Health Education Committee.

The Morning Session was closed after the Election of the Following Officers: Honorary Presidents, Drs. Eliza M. Mosher, Grace Peckham Murray. President, Dr. Helena J. C. Kuhlman, State Hospital, Buffalo, N. Y.; 1st Vice-Pres., Dr. Angennette Parry, New York; 2d Vice-Pres., Josephine Walter, New York; 3d Vice-Pres., Edith Stewart, Hume, N. Y.; Secretary, Dr. Marian Craig Potter, Rochester, N. Y.; Treasurer, Dr. Edith R. Hatch, Buffalo, N. Y.; 1st District Councillor, Dr. S. Josephine Baker, New York; 2d District Councillor, Dr. Anna Craig, King's Park, L. I.; 3d District Councillor, Dr. Julia C. McNutt, Albany, N. Y.; 4th District Councillor, Dr. Julia Kimball Qua, Amsterdam, N. Y.; 5th District Councillor, Dr. Elizabeth McNight,

Oswego, N. Y.; 6th District Councillor, Dr. Sara E. Green, Elmira, N. Y.; 7th District Councillor, Dr. Evaline P. Ballintine, Rochester, N. Y.; 8th District Councillor, Dr. Jane Wall Carroll, Buffalo, N. Y.

The following committees were appointed: Scientific Program, Eleanor Parry, N. Y.; Sarah G. Pierson, Rochester, E. Josephine Baker, N. Y. Legislation, Rosalie Slaughter Morton, N. Y.; Ina V. Burt, Phelps, N. Y.; Cornelia White Thomas, Rochester. Public Health, Matilda K. Wallin, N. Y.; Elizabeth P. Thalberg, Poughkeepsie; Isabelle Thompson Smart, N. Y. Arrangements, M. May Allen, Rochester, Cora Billings Lattin, Buffalo.; Emily Dunning Barringer, N. Y.; Julia D. McNutt, Albany; Grace M. Kimball, Poughkeepsie; Frances Cohan, Yonkers, Helen D. Justin, Castile; Evelyn Baldwin, Rochester. Toastmaster, Grace Peckham Murray, N. Y. Elizabeth T. Corbett, N. Y. was elected Honorary Member and the others who became members were: Christiana M. Greene, Buffalo; S. Augusta Beebe, N. Y.; Jane North Baldwin, Poughkeepsie; Helen Montague, N. Y.; Ethel Blackwood Robinson, N. J.; Eleanor Tomes, N. Y.; A. B. Rice, Nova Scotia; Anna E. Voorhies, Ossining; Rosetta Sherwood Hall, Pyong Yang, Corea; Cornelia E. Brown, N. Y.; Barbara Curtis, Poughkeepsie; Emma A. Selkin, N. Y.; Nadina R. Kavinoky and Lucy A. Kenner, Buffalo; Isabel McMillan, N. Y.; Elizabeth Gillette, Schenectady; Agnes Page, Albany; and Cora Ballard, Brookyn.

In the afternoon there was a Symposium on Sterility:

1. Definition, general remarks, ratio, statistics, by Dr. Katherine S. Munhall; 2. Mechanic causes in women; vaginal, cervical, tubes and ovaries, non-development, by Dr. Angenette M. Parry, N. Y.; 3. Inflammatory causes: Colpismus, endometritis, salpingitis, oophoritis, physical causes, Edith Stewart, Hume, N. Y.; 4. Artificial Impregnation, Dr. Eliza M. Mosher, N. Y.; 5. General Treatment, Dr. Sarah J. McNutt, N. Y.; 6. Operative Procedures, their utility and results, Emily Dunning Barringer, N. Y.

The annual Banquet was held Friday evening at the Hotel Statler. Dr. Ida Bender was a charming toast-master and very gracefully introduced the different speakers. Dr. Grace Peckham Murray, the out-going President, spoke on the Past of the Society and Dr. Helena Kuhlman, the in-coming President, on the "Future." After these more serious speeches, followed those in lighter vein, paraphased on Shakespeare's Seven Ages of Man. Dr. Maude Frye spoke of the Infant. Dr. Marian Craig Potter disposed of the exuberant energies of the "Whining School-Boy." The Lover was charmingly described by Dr. Parry on the ground that one could speak best of that of which one knew the least as the imagination could supply the deficiencies. The Fourth Age arranged to apply to our brother M. D. that we cannot resist the temptation to reproduce it.

"Here's to our brothers who live at their ease
Whose Conscience allows them to do as they please,
Whose fads and whose follies and foolish ideas,
Are brought to us yearly from over the seas
Here's to those brother M.D's.

Here's to them who are eager for Fame
Who want to write Glory all over their name,
With Teddy the Bold they're one and the same,
"Here's hoping they'll win" we gladly exclaim
Here's to those brother M.D's.

And here's to those who have no use for us
Who never can meet us without a cuss
'Tis a fact we deplore, but it might be "Wuss"
It's not worth the least of a bit of fuss
Here's to those brother M.D's.

And here's to—"God bless him"—our noble Ideal
Whose Christian Charity's a quality real,
Whose life and whose love makes all men feel
'Tis a wonderful gift to be able to heal
Here's to that brother M.D."

"And then the Justice" was the topic for Dr. Lettie H. Woodruff of Rochester and Dr. Bender filled in the succeeding toast, also a rearrangement of Shakespeare as "Shifts into? The last scene of all" responded to by Dr. Mosher, Honorary President of the Society and practicing physician for 37 years. Her retrospect of the value of the study of medicine, not only to the world at large, but to the individual student and practitioner was a charming close to the program. Dr. Maude Frye was chairman of the Committee of Arrangements, the other local member being Dr. Edith R. Hatch.

On Thursday evening the Officers and Councillors and Physician's League were entertained at dinner by Dr. Jane Wall Carroll.

---

The Rochester Public Health Association held its annual meeting May 13, 1912. Dr. Henry T. Williams was elected

President; Dr. Williams, Dr. G. I. Bidwell, Dr. John M. Lee, were among the directors chosen; Dr. Montgomery E. Leary was elected a member of the Executive Committee and Dr. Edward G. Whipple continues as Executive Secretary, in charge of the dispensary. The present dispensary building on South Washington St. has been outgrown and plans are under way to secure additional funds and to erect or purchase a larger and more modern building.

---

THE MEDICAL SOCIETY OF THE COUNTY OF MONROE held its regular May meeting at the Hotel Seneca, Rochester, May 21. At the dinner preceeding the meeting, 70 were present, about half the total attendance. Dr. Howard Prince of Ithaca presented a paper on Surgery and the Patient, Dr. John M. Swan on the Importance of Blood Examinations, Dr. E. H. Howard on New Laws Relating to the Mentally Defective.

The most noteworthy business transacted was the preparation of a list of Medical Experts in Insanity. This is a new departure and has for its object the elevation of the standard of medical expert witnesses. The matter was discussed in the May issue of the BUFFALO MEDICAL JOURNAL (page 551) by Dr. Seelye W. Little who deserves the credit of originating the idea. The method has been applied only to alienists as yet and is as follows. A liberal list of nominations is presented by the Board of Censors and a two-thirds vote of the society is necessary to elect. The list will be sent to all judges and lawyers in the county, and though they are free to choose any other person as expert yet the fact that a man is one of the list of experts will presume in his favor. Thus by qualifying our best men the profession will be put into a more dignified position than it has held in the past. It is proposed that lists in the other specialties will ultimately be prepared and that they will be kept revised and additions made as necessary.

The Experts in Insanity for the present are 13 in number as follows:

Dr. E. H. Howard, Dr. E. B. Potter, Dr. I. L. Walker, Dr. W. H. Veeder, Dr. E. P. Ballintine, Dr. M. A. Nickerson, and Dr. Sarah Pierson, all members of the Staff of the Rochester State Hospital, and the following residing in the city—Dr. E. B. Angell, Dr. E. L. Hanes, Dr. W. J. Herriman, Dr. P. W. Neefus, Dr. A. C. Remington and Dr. C. A. Van der Beek.

